# Acetic Acid Enhanced Narrow Band Imaging for the Diagnosis of Gastric Intestinal Metaplasia

**DOI:** 10.1371/journal.pone.0170957

**Published:** 2017-01-30

**Authors:** Jie Sha, Pin Wang, Bingliang Zhu, Minghui Zhu, Xueliang Li, Feng Gao

**Affiliations:** 1 Department of Gastroenterology, the First Affiliated Hospital of Nanjing Medical University, Nanjing, Jiangsu, China; 2 Department of Gastroenterology, Jingjiang People’s Hospital, Jingjiang, Jiangsu, China; 3 Department of Gastroenterology, Drum Tower Clinical Medical School of Nanjing Medical University, Nanjing, Jiangsu, China; University Hospital Llandough, UNITED KINGDOM

## Abstract

Gastric intestinal metaplasia (GIM) is a precancerous lesion of the stomach. The detection of GIM using conventional white-light endoscopy (WLE) is difficult. In this study, we determined whether acetic acid-enhanced narrow band imaging (AA-NBI) improves the detection of GIM. A consecutive cohort of 132 individuals aged 40 years or older was subjected to upper gastrointestinal endoscopy using WLE, NBI and AA-NBI. The ability of the three methods to diagnose GIM in patients was compared. Histological assessment (per-patient and per-biopsy) was used for the accuracy assessment. Sixty-six (50.0%) out of the 132 individuals examined were found to have GIM, of which 44 (66.7%) were diagnosed correctly by NBI (sensitivity 66.7% and specificity 68.2%) and 58 (87.9%) were correctly identified by AA–NBI (sensitivity 87.9% and specificity 68.2%), as compared to only 22 (33.3%) by WLE (sensitivity 33.3% and specificity 28.8%). Therefore, the sensitivity of AA–NBI in the diagnosis of GIM was significantly higher than NBI (p<0.05) and WLE (p < 0.001). Our study indicates that AA-NBI can improve the accuracy of endoscopy-targeted biopsies for GIM.

## Introduction

Gastric cancer is the third leading cause of cancer death worldwide [1]. The detection of early-stage gastric neoplastic lesions may improve survival and avoid complete resection. Gastric cancer onset is considered a multistep process that includes the consecutive development of chronic gastritis followed by mucosal atrophy, gastric intestinal metaplasia (GIM), dysplasia, and finally adenocarcinoma [2]. The surveillance of patients with GIM may therefore lead to the earlier detection of advanced precancerous lesions and gastric cancer [3].

The gold standard for diagnosing GIM remains the histology of biopsy specimens. However, the major limitation of this approach is that GIM exhibits few macroscopic morphological changes, and as a consequence, GIM may readily be missed with random biopsy sampling. Recently, several new endoscopic techniques have been developed to increase the detection of GIM, including chromoendoscopy, autofluorescence imaging, confocal laser endomicroscopy, flexible spectral imaging color enhancement, narrow band imaging (NBI) and magnification endoscopy [4–11]. Currently, there is still no unified standard for chromoendoscopy in the diagnosis of GIM. Moreover, the use of methylene blue carries the risk of causing oxidative DNA damage [12], while autofluorescence imaging, confocal laser endomicroscopy and flexible spectral imaging color enhancement are hard to manipulate. Therefore, these techniques are not generally used in clinical practice.

NBI is an endoscopic imaging technology, which results in the good contrast of surface structures and vascular architecture in the superficial mucosa using blue (400–430 nm) and green (535–565 nm) narrow-band light. These wavelengths are close to the light absorption peaks of hemoglobin [11]. NBI with magnification endoscopy can provide a microscopic image of the mucosal and vascular structures, which are used for the detection of GIM [11, 13].

Acetic acid is a weak acid that breaks the disulfide bonds of glycoproteins of the mucus layer, causing reversible denaturation of the intracellular cytoplasmic proteins. In the columnar epithelium, acetic acid leads to the enhancement of the mucosal architecture and pit-pattern [14–15]. Acetic acid combined with magnification endoscopy or indigo carmine has been used to diagnose gastric neoplasia [14–18]. In this study, we determined whether NBI in combination with acetic acid (AA-NBI) improves the diagnosis of GIM.

## Patients and Methods

### Patients

The present study was performed in 132 consecutive patients who were 40 years or older and required endoscopic examination at the Jingjiang People’s Hospital from February to June 2016. Exclusion criteria were patients with noticeable advanced gastric cancer, previous gastrectomy or partial gastric resection, on-going treatment with antiplatelet medication, anticoagulant medication or nonsteroidal anti-inflammatory drugs, gastrorrhagia and the presence of hemorrhagic diseases. A written informed consent was obtained from all patients before examination. Patients were followed up every two years. This study was approved by the Institutional Research Ethics Committee (IREC) of the Jingjiang People’s Hospital and registered with Chinese Clinical Trial Registry (ChiCTR-DDD-16007999).

### Endoscopic and Biopsy Procedures

**Fig 1:**
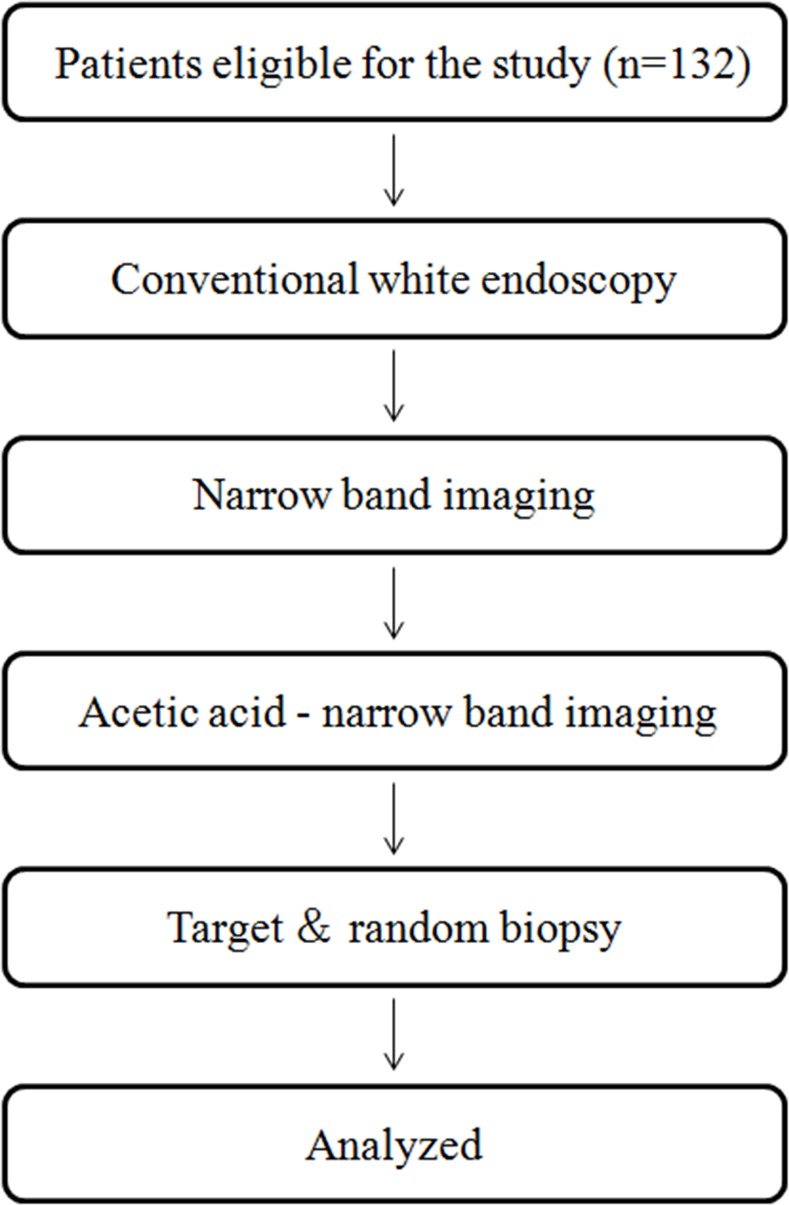
Flow diagram of patient enrollment and endoscopic procedure.

The study flowchart was shown in Fig 1. Conventional white-light endoscopy (WLE), NBI and AA-NBI were performed in all patients by the same endoscopist specializing in NBI and WLE endoscopy during a single procedure with a GIF Q260 endoscope (Olympus Medical Systems, Tokyo, Japan). Because the gastric antrum and angulus are the regions of the highest prevalence of GIM and the acetic acid whitening time is only a few seconds to a few minutes, it is difficult to observe the entire stomach during such a short time. Therefore, we selected the gastric antrum and angulus as the regions for examination in this study. First, the esophagus, stomach and duodenum were carefully examined using WLE. Mucus adhering to the mucosa of the gastric antrum and angulus was washed away as thoroughly as possible. All suspicious antral and angular gastric lesions were photographed. Currently, there are no standard criteria for GIM in WLE; therefore, any abnormal mucosal change, such as localized discoloration and rough areas, was considered to be indicative of GIM lesions in this study.

**Fig 2:**
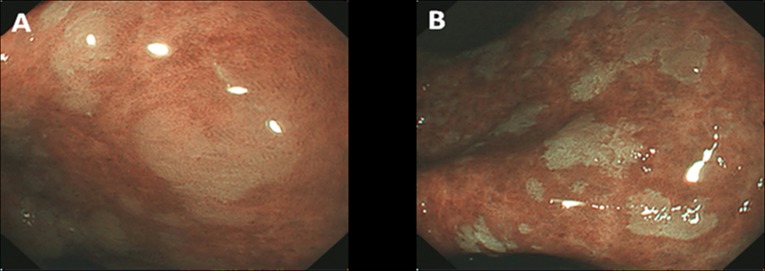
Appearance of the GIM with the lens closing to the lesions. A, the lesion shows bluish-whitish areas with a regular mucosal pattern in the NBI model. B, the lesion shows whitish patches with a regular mucosal pattern in the AA-NBI model.

The endoscopist used the NBI system to carefully observe the gastric antrum and angulus. After found a suspicious lesion, the endoscopist would move the lens close to the lesion to observe the mucosal pattern using no zoomed endoscopy. All suspicious lesions were photographed. NBI suspicious lesions for GIM were defined as bluish-whitish areas with a regular mucosal pattern (Fig 2A). Finally, acetic acid (Zhenjiang white vinegar, from Zhenjiang Vinegar Factory, China) diluted with water (0.6%) was evenly applied to the antrum and angulus areas through the forceps channel in the NBI model. Suspicious GIM lesions were defined as whitish patches with a regular mucosal pattern (Fig 2B). It should mention that, during all these practice, another experienced endoscopist confirmed the lesions simultaneously. The positions of the lesions detected by WLE, NBI or AA-NBI were recorded to ensure the precision of the biopsies obtained. At least one targeted biopsy was separately collected from the endoscopic lesions suspected of GIM by AA-NBI or WLE, and two random biopsies were collected from the antrum and angulus in areas where there were no abnormal findings to serve as controls. If no suspected lesions were identified by AA-NBI, NBI and WLE, three random biopsies were obtained in the antrum and angulus according to the updated Sydney classification [19].

### Histological Assessment

All biopsy specimens were fixed in 4% formalin and embedded in paraffin. The slides were routinely processed with hematoxylin and eosin (H&E) stains. All histologic analyses were performed by an experienced gastrointestinal pathologist who was unaware of the endoscopic results. The histological diagnosis was reported according to the updated Sydney Classification for chronic gastritis and the modified Vienna criteria for neoplasia.

### Statistical Analysis

For the per-patient analysis, the endoscopically suspected lesions in one patient were considered one unit of analysis in this evaluation, although some patients had more than one lesion. Within each patient, only the most severe precancerous grading was evaluated. For example, if a patient had chronic gastritis and GIM, they were classified as GIM. The sensitivity, specificity, positive predictive values, negative predictive values and accuracy for the prediction of GIM in the AA-NBI, NBI and WLE models were calculated using histology as a reference value. For the per-biopsy analysis, the diagnostic accuracy of the targeted biopsies for GIM for the AA-NBI and WLE models were calculated in each specimen. All statistical analyses were performed using Statistical Package for Social Sciences (SPSS 17.0 Package Facility, SPSS Inc., Chicago, Illinois USA). The chi-squared test was used to statistically compare the two groups. A *P*-value less than 0.05 was considered significant.

## Results

### Clinical Characteristics of Patients Involved

From February to June, 2016, a total of 132 eligible patients in Jingjiang People’s Hospital were recruited for this study. The demographics of the patients are summarized in Table 1. The characteristics of the lesions identified in this study are summarized in Table 2. Male patients represented 40.2% (53/132) of the cohort. Macroscopically, a mucosal whitish color was observed in 13 (9.8%) subjects, a mucosal reddish color was observed in 53 (40.2%) subjects, mucosal roughness was observed in 31 (23.5%) subjects and normal mucosa was observed in 35 (26.5%) subjects. In addition, 8.3% (11/132) were current smokers and 12.1% (16/132) of the patients were alcohol consumers.

**Table 1:** The demographics of the study samples.

Variable		n = 132
Age (y)		53.5 (10.1)*
Sex, n (%)	Male	53 (40.2)
	Female	79 (59.8)

*Mean (SD)

**Table 2:** Clinical characteristics of the study samples.

Variable	n = 132
Macroscopic, n (%)	
Whitish	13 (9.8)
Reddish	53 (40.2)
Rough	31 (23.5)
Normal	35 (26.5)
Smoking, n (%)	11 (8.3)
Alcohol drinking, n (%)	16 (12.1)

### Diagnostic Accuracy of Endoscopy for Patients with GIM (Per-Patient Analysis)

**Fig 3:**
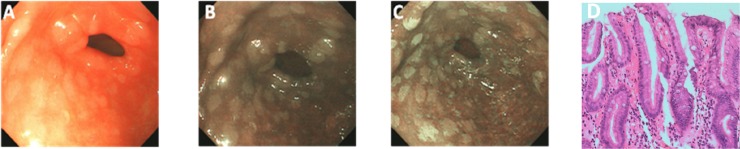
Appearance of intestinal metaplasia in the antrum of the same patient under three different endoscopic models. A, Endoscopic image in WLE shows ash-colored nodular changes. B, After being switched to the NBI model, the lesions exhibit as bluish-whitish areas. C, The clearer whitish patches are observed after sprinkling with acetic acid in the AA-NBI model. D, Targeted biopsy shows intestinal metaplasia of the stomach.

**Fig 4:**
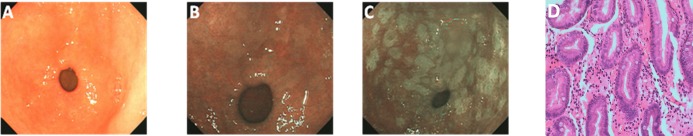
Appearance of intestinal metaplasia in the antrum of the same patient under three different endoscopic models. A, Endoscopic image in WLE shows antrum mucosa is normal. B, After being switched to the NBI model, bluish-whitish areas appear. C, The clearer whitish patches are observed after sprinkling with acetic acid in the AA-NBI model. D, Targeted biopsy shows intestinal metaplasia of the stomach.

**Fig 5:**
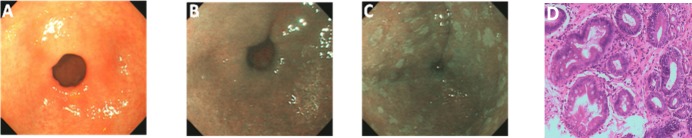
Appearance of intestinal metaplasia in the antrum of the same patient under three different endoscopic models. A, Endoscopic image in WLE shows antrum mucosa is normal. B, After being switched to the NBI model, no obvious bluish-whitish areas appear. C, The whitish patches are observed after sprinkling with acetic acid in the AA-NBI model. D, Targeted biopsy shows intestinal metaplasia of the stomach.

As described before, GIM primarily showed bluish-whitish areas in the NBI model (Figs 3B and 4B), and whitish patches in the AA-NBI model (Figs 3C, 4C and 5C). Only in one patient low-grade intraepithelial neoplasia was detected. No patients with high-grade intraepithelial neoplasia or gastric cancer were identified. Among all the patients examined, 66 (50.0%) were histologically confirmed with GIM.

#### Detection of GIM by WLE versus AA-NBI

Of the 66 patients with historically confirmed GIM, GIM was detected in 20 (30.3%) by both WLE and AA-NBI, whereas GIM was detected in 38 (57.6%) solely by AA-NBI and in 2 (3.0%) by WLE. GIM was detected in the remaining 6 (9.1%) patients by random biopsy (Table 3).

**Table 3:** Patients with histologically confirmed diagnosis of GIM.

Detection method	Histologically confirmed
WLE & AA-NBI	20
AA-NBI	38
WLE	2
Random biopsies	6

During the endoscopic examination using AA–NBI, whitish patches were identified in 79 patients, of which 58 cases of GIM were confirmed by histology. There were one case of low-grade intraepithelial neoplasia and 20 other cases of inflammation. WLE identified 97 patients with mucosal abnormal areas, of which 22 cases were confirmed with GIM by histology. Of the remaining 75 cases, 47 showed inflammation, and 28 showed inflammation in WLE targeted biopsies while showed GIM in AA-NBI targeted biopsies. Ten cases showed normal in WLE, but whitish patches in AA-NBI, and these patients were showed GIM by targeted biopsies.

Based on the above results, WLE had a sensitivity of 33.3%, a specificity of 28.8%, a positive predictive value of 31.9%, a negative predictive value of 30.2% and a accuracy of 31.1% in the diagnosis of GIM. In comparison, AA-NBI showed a sensitivity of 87.9%, a specificity of 68.2%, a positive predictive value of 73.4%, a negative predictive value of 84.9% and a accuracy of 78.0% for the diagnosis of GIM. The sensitivity, specificity, positive predictive values, negative predictive values and accuracy of AA-NBI were all significantly higher than those of WLE (P<0.001) (Table 4).

**Table 4:** Diagnostic accuracy of endoscopy in patients with GIM by WLE, NBI and AA-NBI.

GIM	Sensitivity	Specificity	PPV	NPV	Accuracy
WLE	33.3%(22/66)^a^	28.8%(19/66)^a^	31.9%(22/69)^a^	30.2%(19/63)^a^	31.1%(41/132)^a^
NBI	66.7%(44/66)^b^	68.2%(45/66)	67.7%(44/65)	67.2%(45/67)^b^	67.4%(89/132)
AA-NBI	87.9%(58/66)	68.2%(45/66)	73.4%(58/79)	84.9%(45/53)	78.0%(103/132)

PPV positive predictive value, NPV negative predictive value.

^a^AA-NBI versus WLE: *p* <0.001

^b^AA-NBI versus NBI: P<0.05.

#### Detection of GIM by NBI versus AA-NBI

Of the 66 GIM patients diagnosed by histology, obvious bluish-whitish areas in the NBI model were not observed in 14 (21.2%) patients, but after the administration of acetic acid, whitish patches appeared, and targeted biopsies in such areas confirmed GIM. Overall, in the diagnosis of GIM, NBI had a sensitivity of 66.7%, a specificity of 68.2%, a positive predictive value of 67.7%, a negative predictive value of 67.2% and a accuracy of 67.4% in the diagnosis of GIM. The sensitivity, negative predictive value of AA-NBI were significantly higher than those of NBI (*p*<0.05) (Fig 5, Table 4).

### Diagnostic Accuracy of Targeted Biopsies for GIM (Per-biopsy Analysis)

For AA-NBI, a total of 182 targeted biopsies were obtained. Of these, 106 specimens were histologically diagnosed as GIM, two were low-grade intraepithelial neoplasia, and 74 were chronic inflammation. For WLE, a total of 192 targeted biopsies were obtained. Of these, 41 specimens were diagnosed as GIM and 151 were chronic inflammation. Therefore, for the per-biopsy analysis, AA-NBI with targeted biopsies had a significantly greater diagnostic ability for GIM compared with WLE, with 58.2% (106/182) versus 21.4% (41/192) (P<0.001).

## Discussion

GIM is an important risk factor for intestinal gastric cancer [2]. However, the diagnosis of GIM using conventional WLE is unreliable because GIM usually appears in flat mucosa with few macroscopic morphological changes and occurs multifocally [4, 20, 21].

Nomura H et al. [22] defined GIM as a lesion appearing as ash-colored nodular changes as observed in conventional WLE. The sensitivity and specificity of this diagnostic procedure were reported to be 6% and 98%, respectively. Lim JH et al. [23] defined GIM as the presence of whitish plaques, patches, or homogeneous whitish discoloration on the gastric mucosa and reported that the sensitivity and specificity were 24.0% and 91.9% for the lesions in the antrum, respectively, and 24.2% and 88.0% for the lesions in the body, respectively. These results from various hospitals showed that the sensitivity of conventional WLE for the diagnosis of GIM is very low. Therefore, there is an urgency to increase the sensitivity of endoscopic diagnosis of GIM.

The NBI technique is based on a modification of the spectral characteristics of the optical filter in the light source, resulting in improved visibility of the mucosal structures [11]. It is a unique sequential electronic endoscopy system. One of the greatest advantages of this system is its capacity of visualizing the minute mucosal surface without chromoendoscopy. It was previously reported that NBI alone or combined with magnification endoscopy (ME) can reveal mucosal lesions, and NBI-ME is more useful for diagnosing abnormal patterns than NBI without magnification [24]. However, magnifying endoscopes have not been widely used due to their complicated operation.

The method of sprinkling acetic acid to observe the specialized columnar epithelium of Barrett’s esophagus was originally reported by Guelrud et al.[25]. There were several previous reports of acetic acid enhanced magnifying endoscopy for the diagnosis of gastric neoplasia [14,15]. The transient white coloration of the epithelial surface, which occurs after the spraying of acetic acid, is a phenomenon of increased opacity. This corresponds to the reversible alteration of the tertiary structures of cellular proteins upon applying the acetic acid. Several reports also described acetic acid with indigo carmine for the diagnosis of early gastric cancer [16–18].

In our study, we firstly combined NBI with acetic acid (AA-NBI) for the diagnosis of GIM. We observed the following: 1. Ash-colored nodular lesions in WLE appear bluish-whitish in the NBI model alone, and whitish patches are observed after sprinkling acetic acid (AA-NBI); 2. No ash-colored nodular changes are observed in WLE, and the lesions also appear bluish-whitish in the NBI model; however, the clearer whitish patches are observed after sprinkling with acetic acid; and 3. The area shows no abnormal change in WLE and NBI, but becomes a whitish patch in the AA-NBI.

Our study showed that AA-NBI allowed us to detect GIM in patients with a sensitivity of 87.9%, a specificity of 68.2%, a positive predictive value of 73.4%, a negative predictive value of 84.9% and an accuracy of 78.0%, all of which were significantly higher than those of WLE.

Our study also showed that bluish-whitish areas were not found in the NBI model in some GIM subjects, whereas whitish patches appeared in the AA-NBI. Overall, compared to NBI, the sensitivity for the detection of GIM was increased by 21.2% for AA-NBI, and the negative predictive value for the latter was also higher than that of NBI. The sensitivity, specificity, positive predictive value, and negative predictive value in our study using NBI are close to those published by Capelle et al. [10], who achieved a sensitivity of 71%, a specificity of 58%, a positive predictive value of 65.0% and a negative predictive value of 65.0% using NBI for the diagnosis of GIM.

Our study had some limitations. First, the study was performed at a single center only, although it was necessary for our early stage comparison of the three relevant techniques. Obviously larger multicenter prospective studies will be warranted to validate the findings from the current study. Second, the endoscopic procedures for WLE and AA-NBI were performed by the same endoscopist for consistence; however, the detection of GIM using AA-NBI may cause bias because of the previous WLE observations. Third, the antrum and angulus were selected in this study because they are the regions of the highest prevalence of intestinal metaplasia. A future study is necessary to confirm whether AA-NBI accurately detects GIM in the whole stomach.

## Conclusions

The whitish patches observed in the gastric mucosa with AA-NBI are highly accurate indicators for GIM. AA-NBI can improve the accuracy of endoscopy-targeted biopsies for GIM.

## Supporting Information

S1 Checklist Trend checklist 1. Trend statement checklist.
(PDF)

S2 Checklist Trend checklist 2. Trend statement checklist.
(PDF)

S3 Checklist Trend checklist 3. Trend statement checklist.
(PDF)

S1 File Study protocol Chinese. Study protocol of acetic acid enhanced narrowband imaging for the diagnosis of gastric lesions.
(PDF)

S2 File Study protocol. Study protocol of acetic acid enhanced narrow band imaging for the diagnosis of gastric lesions.
(PDF)

S3 File Minimal data. Minimal data set.
(PDF)
