# Supplementary material for: Acetic Acid Enhanced Narrow Band Imaging for the Diagnosis of Gastric Intestinal Metaplasia
Source: PLoS One. 2017 Jan 30;12(1):e0170957. doi: 10.1371/journal.pone.0170957 (PMC5279783; doi:10.1371/journal.pone.0170957)
Supplement: S2 Checklist — Trend statement checklist. (PDF) [file pone.0170957.s002.pdf]

TREND Statement Checklist

Blinding (masking)	9	• Whether or not participants, those administering the interventions, and those assessing the outcomes were blinded to study condition assignment; if so, statement regarding how the blinding was accomplished and how it was assessed.	√	6
Unit of Analysis	10	• Description of the smallest unit that is being analyzed to assess intervention effects (e.g., individual, group, or community)	√	6
		• If the unit of analysis differs from the unit of assignment, the analytical method used to account for this (e.g., adjusting the standard error estimates by the design effect or using multilevel analysis)		
Statistical Methods	11	• Statistical methods used to compare study groups for primary methods outcome(s), including complex methods of correlated data	√	7
		• Statistical methods used for additional analyses, such as a subgroup analyses and adjusted analysis		
		• Methods for imputing missing data, if used		
		• Statistical software or programs used	√	7
**Results**				
Participant flow	12	• Flow of participants through each stage of the study: enrollment, assignment, allocation, and intervention exposure, follow-up, analysis (a diagram is strongly recommended)		
		o Enrollment: the numbers of participants screened for eligibility, found to be eligible or not eligible, declined to be enrolled, and enrolled in the study		
		o Assignment: the numbers of participants assigned to a study condition	√	7
		o Allocation and intervention exposure: the number of participants assigned to each study condition and the number of participants who received each intervention	√	7
		o Follow-up: the number of participants who completed the follow-up or did not complete the follow-up (i.e., lost to follow-up), by study condition		
		o Analysis: the number of participants included in or excluded from the main analysis, by study condition	√	7
		• Description of protocol deviations from study as planned, along with reasons		
Recruitment	13	• Dates defining the periods of recruitment and follow-up	√	7
Baseline Data	14	• Baseline demographic and clinical characteristics of participants in each study condition	√	7-8
		• Baseline characteristics for each study condition relevant to specific disease prevention research		
		• Baseline comparisons of those lost to follow-up and those retained, overall and by study condition		
		• Comparison between study population at baseline and target population of interest		
Baseline equivalence	15	• Data on study group equivalence at baseline and statistical methods used to control for baseline differences		
