# Supplementary material for: Acetic Acid Enhanced Narrow Band Imaging for the Diagnosis of Gastric Intestinal Metaplasia
Source: PLoS One. 2017 Jan 30;12(1):e0170957. doi: 10.1371/journal.pone.0170957 (PMC5279783; doi:10.1371/journal.pone.0170957)
Supplement: S1 File — Study protocol of acetic acid enhanced narrowband imaging for the diagnosis of gastric lesions. (PDF) [file pone.0170957.s004.pdf]

Study protocol of acetic acid enhanced narrow band imaging for the diagnosis of gastric lesions

醋酸联合窄带成像技术诊断胃黏膜病变的临床研究方案

研究单位：　　靖江市人民医院

承担科室：　　消化科

联系电话：　　0523-84995951

版本号：　　V2.0

版本日期：　　2014-12-10

一、 立项依据

胃癌是严重危害人类健康的疾病，其死亡率仅次于肺癌居全球癌症死亡率的第二位[1]，胃癌的预后直接与诊断时的分期有关[2-3]，早期胃癌术后5年生存率 ＞ 90%[4-5]。因此降低胃癌死亡率的关键在于早期诊断早期治疗。胃癌的发生发展是一个多因素、多阶段的过程，由正常胃粘膜上皮变为早期胃癌，经历慢性萎缩性胃炎、肠上皮化生、不典型增生多个阶段，因此对慢性萎缩性胃炎、肠上皮化生、不典型增生进行随访监测有利于提高早期胃癌的发现率。目前胃镜结合病理检查仍诊断胃黏膜病变的金标准。但胃黏膜病变在普通胃镜下并无特异性表现，因此胃镜下如何发现病灶并准确活检成为内镜医生必需解决的关键问题。

内镜下检查时喷洒醋酸，最先是由Guelrud等[6]把该项技术用于消化道粘膜病变的检测。后来Yagi等[7]和Tanaka等[8]联合放大内镜应用于胃癌及癌前病变的检测。醋酸可以使上皮细胞蛋白质的三级结构发生可逆性改变，黏膜表面出现一过性白化，从而增加了病变组织和正常组织的对比度。Yamashita等[9]和Sakai等[10]报道醋酸联合靛胭脂染色可提高早期胃癌的发现率和诊断率。我们研究发现醋酸联合靛胭脂染色亦有助于提高上皮内瘤变及肠上皮化生的靶向活检率[11]。

窄带成像内镜（narrow band imaging，NBI）是利用滤光器过滤掉内镜光源所发出的红蓝绿光波中的宽带光谱，仅留下窄带光谱，从而显示黏膜表层的毛细血管和黏膜表面腺管开口形态的一种内镜检查技术。但由于窄带成像技术采用了窄带光源，视野很暗，目前认为，单独应用窄带成像技术对胃异常黏膜的观察优势并不明显，所以NBI常和放大内镜联合使用以使黏膜表面形态显示的更为清楚，但目前在我国窄带成像内镜较为普及，而放大内镜仅集中在国内少数大型医院，因而这一技术很难得以推广。

本研究我们将醋酸联合窄带成像技术应用于胃黏膜病变的诊断。我们前期

研究发现在窄带成像模式下喷洒醋酸可使得病变的表面形态结构显示得更清楚。该研究为胃黏膜病变的诊断提供了一种新的内镜检查方法，具有较好的临床应用前景。

参考文献

[1] Roder DM. The epidemiology of gastric cancer. Gastric Cancer. 2002;5 Suppl 1:5-11.

[2] Tanabe S, Koizumi W, Mitomi H, et al. Clinical outcome of endoscopic aspiration mucosectomy for early stage gastric cancer. Gastrointest Endosc. 2002;56:708-713.

[3] Ono H. Early gastric cancer: diagnosis, pathology, treatment techniques and treatment outcomes. Eur J Gastroenterol Hepatol. 2006;18:863-866.

[4] Onodera H, Tokunaga A, Yoshiyuki T, et al. Surgical outcome of 483 patients with early gastric cancer: prognosis, postoperative morbidity and mortality, and gastric remnant cancer. Hepatogastroenterology. 2004;51:82-85.

[5] Kikuchi S, Katada N, Sakuramoto S, et al. Survival after surgical treatment of early gastric cancer: surgical techniques and long-term survival. Langenbecks Arch Surg. 2004;389:69-74.

[6] Guelrud M, Herrera I. Acetic acid improves identification of remnant islands of Barrett's epithelium after endoscopic therapy. Gastrointest Endosc. 1998;47:512-515.

[7] Yagi K, Aruga Y, Nakamura A, et al. The study of dynamic chemical magnifying endoscopy in gastric neoplasia. Gastrointest Endosc. 2005;62:963- 969.

[8] Tanaka K, Toyoda H, Kadowaki S, et al. Surface pattern classification by enhanced-magnification endoscopy for identifying early gastric cancers. Gastrointest Endosc. 2008;67:430-437.

[9] Yamashita H, Kitayama J, Ishigami H, et al. Endoscopic instillation of indigo

carmine dye with acetic acid enables the visualization of distinct margin of superficial gastric lesion; Usefulness in endoscopic treatment and diagnosis of gastric cancer. Dig Liver Dis. 2007;39:389-391.
[10] Sakai Y, Eto R, Kasanuki J, et al. Chromoendoscopy with indigo carmine dye added to acetic acid in the diagnosis of gastric neoplasia: a prospective comparative study. Gastrointest Endosc. 2008,68:635-641.
[11] 沙杰，李学良，施瑞华等. 醋酸联合靛胭脂染色诊断早期胃癌及癌前病变的临床价值.中华消化内镜杂志.2010;27:644-646.

二、 项目的研究内容、研究目标、关键技术、创新之处

1）研究内容

探讨内镜下醋酸联合窄带成像技术诊断胃黏膜病变的临床价值。

具体内容如下：

（1） 纳入因各种消化道症状在我科接受常规胃镜检查，发现以下至少 1 种病变者：⑴黏膜粗糙；⑵黏膜色泽发生改变；⑶黏膜下血管网模糊、紊乱或消失；⑷黏膜局部隆起；(5)浅表糜烂或溃疡；⑹局部胃壁蠕动差。常规胃镜检查即可基本明确诊断的胃息肉、胃癌患者、胃出血患者、有凝血障碍或血小板$<50\times10^9$/L 者不纳入。

（2） 胃镜检查前常规服用利多卡因胶浆，发现有胃黏膜异常者尽量冲净胃黏膜表面的黏液及附着物，撤换至窄带成像模式保存图像，窄带成像模式下喷洒 0.6%醋酸后保存图像，行组织病理学检查。

（3） 对醋酸联合窄带成像技术后上皮下微血管结构及粘膜表面的微形态结构进行分型，总结不同特点对于胃黏膜病变的诊断价值，并与普通窄带成像技术进行比较。分别计算其敏感性、特异性、阳性预测

值、阴性预测值，对比分析。

2）研究目标

探讨该方法对内镜下判断胃黏膜病变的有效性，为临床推广应用提供依据。

3）关键技术

（1） 内镜下醋酸联合窄带成像技术。

（2） 标本HE染色，组织病理学检查。

4）创新之处

本课题将醋酸联合窄带成像技术应用于胃黏膜病变的诊断。

三、研究方法及技术路线

1）研究方法

（1）纳入因各种消化道症状在我科接受常规胃镜检查，发现以下至少 1 种病变者：(1)黏膜粗糙；(2)黏膜色泽发生改变；(3)黏膜下血管网模糊、紊乱或消失；(4)黏膜局部隆起；(5)浅表糜烂或溃疡；(6)局部胃壁蠕动差。常规胃镜检查即可基本明确诊断的胃息肉、胃癌患者、胃出血患者、有凝血障碍或血小板$<50\times10^9$/L 者不纳入。

（2）胃镜检查前常规服用利多卡因胶浆，发现有胃黏膜异常者尽量冲净胃黏膜表面的黏液及附着物，撤换至窄带成像模式保存图像，窄带成像模式下喷洒 0.6%醋酸后保存图像，行组织活检。

（3）标本行 HE 染色，由同一位经验丰富的病理科医师读片。

（4）对醋酸联合窄带成像技术后上皮下微血管结构及粘膜表面的微形态结构进行分型，总结不同特点对于胃黏膜病变的诊断价值，并与普通窄带成像技术进行比较。分别计算其敏感性、特异性、阳性预测值、阴性预测值，对比分析。应用 SPSS17.0 分析软件，行 χ^2 检验，$P<0.05$ 有统计学意义。

2）技术路线图

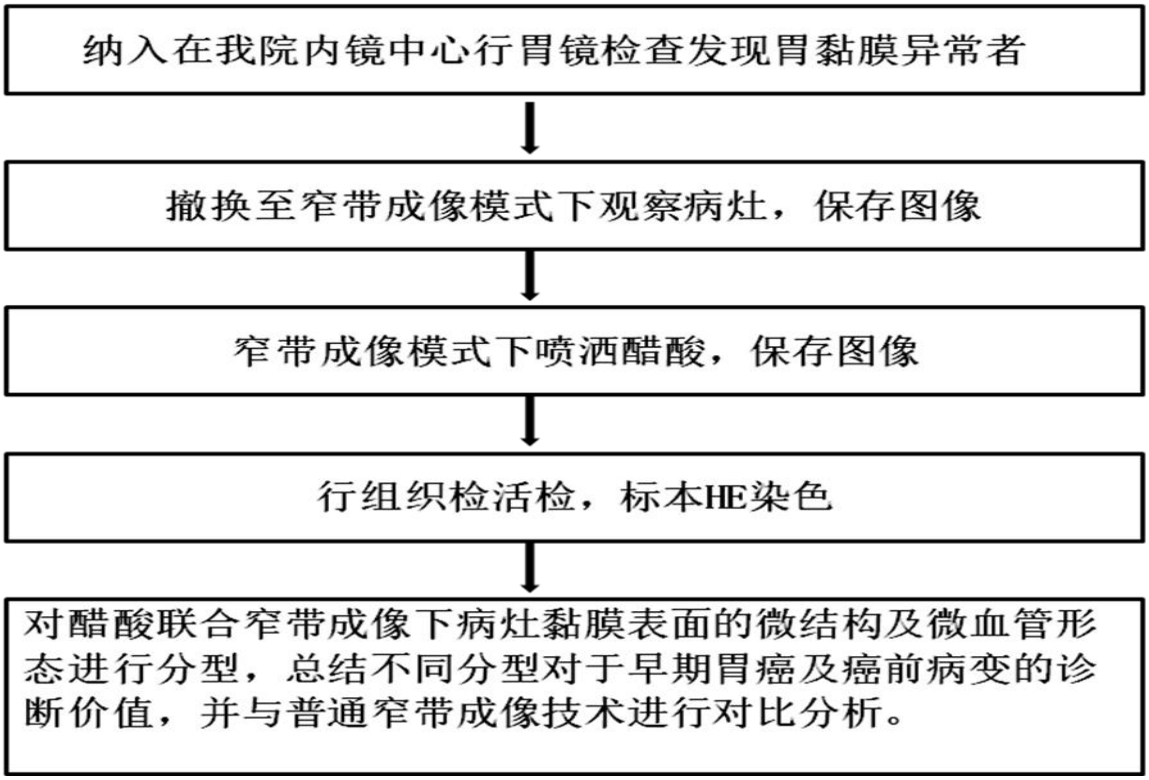

四、研究工作的年度计划和预期研究结果

1) 年度计划

2015年1月—2016年1月　购买相关试剂、文献检索等。

2016年2月—2017年2月　完成相关实验，收集整理数据，撰写论文。

2017年3月—2018年1月　整理总结实验资料，发表论文，完成结题报告，申请成果鉴定。

2) 预期研究结果

醋酸联合窄带成像技术能够清楚地显示上皮下微血管结构及粘膜表面的微形态结构，从而提高胃黏膜病变的靶向活检率。
