# Supplementary material for: Acetic Acid Enhanced Narrow Band Imaging for the Diagnosis of Gastric Intestinal Metaplasia
Source: PLoS One. 2017 Jan 30;12(1):e0170957. doi: 10.1371/journal.pone.0170957 (PMC5279783; doi:10.1371/journal.pone.0170957)
Supplement: S2 File — Study protocol of acetic acid enhanced narrow band imaging for the diagnosis of gastric lesions. (PDF) [file pone.0170957.s005.pdf]

Study protocol of acetic acid enhanced narrow band imaging for the diagnosis of gastric lesions

Study institution: Jingjiang people hospital

Take department: Gastroenterology

Telephone: +860523-84995951

Version number: V2.0

Version date: 2014-12-10

1. Project basis

Gastric cancer is the second leading cause of cancer death in the world.[1] The detection of early-stage gastric neoplastic lesions is associated with improved survival and the potential for complete resection.[2-3] Gastric cancer is a multistep process that includes the consecutive development of chronic gastritis followed by mucosal atrophy, Gastric intestinal metaplasia, dysplasia, and finally adenocarcinoma.[4] The surveillance of patients with precancerous lesion may therefore lead to the earlier detection of gastric cancer. The gold standard for diagnosing gastric lesions remains the histology of biopsy specimens. The major limitation of this approach is that gastric lesions show few macroscopic morphological changes, and thus, may be missed with random biopsy sampling. Therefore, it is the key problem for endoscopic doctors to find the lesion and precise biopsy.

Originally, the method of sprinkling acetic acid to delineate lesions was reported by Guelrud et al. [5] Yagi et al. [6] and Tanaka et al.[7] then combined acetic acid with magnification endoscopy for the assessment of gastric neoplasms. The transient white coloration of the epithelial surface, which occurs after the spraying of acetic acid, is a consequence of increased opacity. This corresponds to reversible alteration of tertiary structures of cellular proteins. Yamashita et al. [8] and Sakai et al. [9] reported acetic acid indigo carmine mixture method was useful for detecting the early gastric cancer. Our previous study found that indigo carmine added to acetic acid helps to improve the target biopsy rat in intraepithelial neoplasia and intestinal metaplasia. [10]

Narrow band imaging (NBI) is an endoscopic imaging technology, which results in the good contrast of surface structures and vascular architecture in the superficial mucosa using blue and green narrow-band light. [11] However, due to the large stomach cavity, the vision is always dark, so it is not obvious advantage to observe the gastric lesions in NBI. NBI with magnification endoscopy can provide a microscopic image of the mucosal and vascular structures, which are useful for the detection of gastric lesions. But magnification is not popular in china, so it is not practical.

In this study, we combine acetic acid with narrow band imaging in the diagnosis

of gastric mucosal lesions. Our previous study found the surface morphology of the lesion can be clearly displayed in acetic acid enhanced narrow band imaging. This study provides a new method in the diagnosis of gastric lesions, and has a good clinical application prospect.

References

[1] Roder DM. The epidemiology of gastric cancer. Gastric Cancer. 2002;5 Suppl 1:5-11.

[2] Tanabe S, Koizumi W, Mitomi H, et al. Clinical outcome of endoscopic aspiration mucosectomy for early stage gastric cancer. Gastrointest Endosc. 2002;56:708-713.

[3] Ono H. Early gastric cancer: diagnosis, pathology, treatment techniques and treatment outcomes. Eur J Gastroenterol Hepatol. 2006;18:863-866.

[4] P. Correa, Human gastric carcinogenesis: a multistep and multifactorial process--First American Cancer Society Award Lecture on Cancer Epidemiology and Prevention. Cancer research 52, 6735 (Dec 15, 1992).

[5] Guelrud M, Herrera I. Acetic acid improves identification of remnant islands of Barrett's epithelium after endoscopic therapy. Gastrointest Endosc. 1998;47:512-515.

[6] Yagi K, Aruga Y, Nakamura A, et al. The study of dynamic chemical magnifying endoscopy in gastric neoplasia. Gastrointest Endosc. 2005;62:963- 969.

[7] Tanaka K, Toyoda H, Kadowaki S, et al. Surface pattern classification by enhanced-magnification endoscopy for identifying early gastric cancers. Gastrointest Endosc. 2008;67:430-437.

[8] Yamashita H, Kitayama J, Ishigami H, et al. Endoscopic instillation of indigo carmine dye with acetic acid enables the visualization of distinct margin of superficial gastric lesion; Usefulness in endoscopic treatment and diagnosis of gastric cancer. Dig Liver Dis. 2007;39:389-391.

[9] Sakai Y, Eto R, Kasanuki J, et al. Chromoendoscopy with indigo carmine dye added to acetic acid in the diagnosis of gastric neoplasia: a prospective comparative study. Gastrointest Endosc. 2008,68:635-641.

[10] Jie Sha, Xueliang li, Ruihua Shi et al. Clinical application value of acetic acid and indigo carmine staining in diagnosis of early gastric cancer and precancerous lesion. Chin J Dig Endosc. 2010,27:644-646.

[11] J. K. An et al. Marginal turbid band and light blue crest, signs observed in

magnifying narrow-band imaging endoscopy, are indicative of gastric intestinal metaplasia. BMC gastroenterology 12, 169 (2012).

2. Study contents, study objective, key technologies, innovations

1) Study contents: To investigate the clinical value of acetic acid enhanced narrow band imaging for the diagnosis of gastric lesions.

   (1) Inclusion criteria are mucosa appeared rough, color of the mucosa altered, capillary network of submucosa blurred or vanished, regional mucosa elevated, superficial erosion or ulceration, passive peristalsis of partially gastric wall. Exclusion criteria are patients with obvious advanced gastric cancer and gastric polyp, treatment with antiplatelet medication, platelet< $50*10^9$, gastrorrhagia and the presence of hemorrhagic diseases.

   (2) First, Lidocaine is taken orally before endoscopy. Mucus adhering to the mucosa of the gastric is washed away as thoroughly as possible. All suspicious lesions are photographed in conventional endoscopy. Then, the endoscopist used the NBI model. All suspicious lesions are photographed. Finally, commercial acetic acid diluted with water (0.6%) is evenly applied in the NBI model. Suspicious lesions are photographed. The histopathology is performed.

   (3) All biopsy specimens are fixed in 4% formalin and embedded in paraffin. Next, the slides are routinely stained with hematoxylin and eosin. All histologic analyses are performed by an experienced GI pathologist.

   (4) The microvascular and microstructure are analyzed and typed in acetic enhanced narrow band imaging model. Sum up the diagnostic value of different feature for gastric mucosal lesions, and compare with narrow band imaging and conventional endoscopy. The sensitivity, specificity, positive predictive value and negative predictive are calculated respectively. The results are compared and analyzed.

2) Study objective: To investigate the effectiveness of this method in the diagnosis of gastric lesions, and provide basis for clinical application.

3) Key technologies: Acetic acid combined with narrow band imaging, Hematoxylin and eosin staining, Histopathology.
4) Innovations: In the study, we firstly combine acetic acid with narrow band imaging for the diagnosis of gastric lesions.

3. Study methods and study flowchart

1) Study methods

(1) Inclusion criteria are mucosa appeared rough, color of the mucosa altered, capillary network of submucosa blurred or vanished, regional mucosa elevated, superficial erosion or ulceration, passive peristalsis of partially gastric wall. Exclusion criteria are patients with obvious advanced gastric cancer and gastric polyp, treatment with antiplatelet medication, platelet< $50*10^9$, gastrorrhagia and the presence of hemorrhagic diseases.

(2) First, Lidocaine is taken orally before endoscopy. Mucus adhering to the mucosa of the gastric is washed away as thoroughly as possible. All suspicious lesions are photographed in conventional endoscopy. Then, the endoscopist used the NBI model. All suspicious lesions are photographed. Finally, commercial acetic acid diluted with water (0.6%) is evenly applied in the NBI model. Suspicious lesions are photographed. The histopathology is performed.

(3) All biopsy specimens were fixed in 4% formalin and embedded in paraffin. Next, the slides were routinely stained with hematoxylin and eosin. All histologic analyses are performed by an experienced GI pathologist.

(4) The microvascular and microstructure are analyzed and typed in acetic enhanced narrow band imaging model. Sum up the diagnostic value of different feature for gastric mucosal lesions, and compare with narrow band imaging and conventional endoscopy. The sensitivity, specificity, positive predictive value and negative predictive are calculated respectively. The results are compared and analyzed. All statistical

analyses were performed using Statistical Package for Social Sciences (SPSS 17.0 Package Facility, SPSS Inc., Chicago, Illinois USA). The chi-squared test was used to statistically compare the two groups. A *P*-value less than 0.05 is considered significant.

2) Study flowchart

Include the patients who are willing to examine with NBI

↓

Suspicious lesions are photographed in conventional endoscopy. Then, use the NBI model. All suspicious lesions are photographed.

↓

Suspicious lesions are photographed in conventional endoscopy. Then, use the NBI model. All suspicious lesions are photographed.

↓

Commercial acetic acid diluted with water (0.6%) is evenly applied in the NBI model. Suspicious lesions are photographed.

↓

The histopathology is performed

↓

The microvascular and microstructure are analyzed and typed in acetic enhanced narrow band imaging model. Sum up the diagnostic value of different feature for gastric mucosal lesions, and compare with narrow band imaging and conventional endoscopy.

4. Annual plan and expected results

1) Annual plan

2015.01-2016.01 Purchase related reagents, literature search, et al.

2016.02-2017.02 Complete experiments, Collect data, Write paper.

2017.03-2018.01 Publish paper, Complete reports, Appraisal the study.

2) Expected results

Acetic acid enhanced narrow band imaging can show the microvascular and microstructure of gastric mucosa clearly, and is useful for improving the accuracy of endoscopy-targeted biopsies.
