# Supplementary material for: Acetic Acid Enhanced Narrow Band Imaging for the Diagnosis of Gastric Intestinal Metaplasia
Source: PLoS One. 2017 Jan 30;12(1):e0170957. doi: 10.1371/journal.pone.0170957 (PMC5279783; doi:10.1371/journal.pone.0170957)
Supplement: S3 File — Minimal data set. (PDF) [file pone.0170957.s006.pdf]

Minimal data set

Patient	Sex	Age (y	WLE	NBI	AA-NBI	Location	histological diagnosis
1	Male	48	Whitish	bluish-whitish area	whitish patches	antrum and angul	intestinal metaplasia(AA-NBI,WLE)
2	Male	76	Whitish	bluish-whitish area	whitish patches	antrum	intestinal metaplasia(AA-NBI,WLE)
3	Male	43	Whitish	bluish-whitish area	whitish patches	antrum	intestinal metaplasia(AA-NBI,WLE)
4	Male	58	Whitish	bluish-whitish area	whitish patches	antrum	intestinal metaplasia(AA-NBI,WLE)
5	Male	55	Whitish	bluish-whitish area	whitish patches	antrum	intestinal metaplasia(AA-NBI,WLE)
6	Male	51	Reddish	bluish-whitish area	whitish patches	antrum and angul	intestinal metaplasia(AA-NBI)
7	Male	62	Reddish	bluish-whitish area	whitish patches	antrum and angul	intestinal metaplasia(AA-NBI,WLE)
8	Male	53	Reddish	bluish-whitish area	whitish patches	antrum	intestinal metaplasia(AA-NBI)
9	Male	62	Reddish	bluish-whitish area	whitish patches	antrum	intestinal metaplasia(AA-NBI,WLE)
10	Male	73	Reddish	bluish-whitish area	whitish patches	antrum	intestinal metaplasia(AA-NBI,WLE)
11	Male	48	Rough	bluish-whitish area	whitish patches	antrum and angul	intestinal metaplasia(AA-NBI,WLE)
12	Male	52	Rough	bluish-whitish area	whitish patches	antrum and angul	intestinal metaplasia(AA-NBI,WLE)
13	Male	48	Rough	bluish-whitish area	whitish patches	antrum	intestinal metaplasia(AA-NBI,WLE)
14	Male	62	Rough	bluish-whitish area	whitish patches	antrum	intestinal metaplasia(AA-NBI,WLE)
15	Male	60	Reddish	bluish-whitish area	whitish patches	antrum	intestinal metaplasia(AA-NBI,WLE)
16	Male	51	Reddish	bluish-whitish area	whitish patches	antrum	low-grade intraepithelial neoplasia
17	Male	43	Whitish	bluish-whitish area	whitish patches	antrum	inflammation
18	Male	61	Rough	bluish-whitish area	whitish patches	antrum and angul	inflammation
19	Male	40	Rough	bluish-whitish area	whitish patches	antrum and angul	intestinal metaplasia(AA-NBI)
20	Male	52	Reddish	Normal	whitish patches	antrum	inflammation
21	Male	50	Reddish	bluish-whitish area	whitish patches	antrum	intestinal metaplasia(AA-NBI)
22	Male	57	Reddish	bluish-whitish area	whitish patches	antrum	intestinal metaplasia(AA-NBI)
23	Male	45	Reddish	bluish-whitish area	whitish patches	antrum	intestinal metaplasia(AA-NBI)
24	Male	72	Reddish	bluish-whitish area	whitish patches	antrum	intestinal metaplasia(AA-NBI)
25	Male	50	Reddish	bluish-whitish area	whitish patches	antrum	intestinal metaplasia(AA-NBI)
26	Male	52	Rough	Normal	whitish patches	antrum	intestinal metaplasia(AA-NBI)
27	Male	40	Rough	Normal	whitish patches	antrum and angul	intestinal metaplasia(AA-NBI)
28	Male	43	Normal	Normal	whitish patches	angulus	intestinal metaplasia(AA-NBI)
29	Male	44	Reddish	Normal	whitish patches	antrum	inflammation
30	Male	61	Reddish	Normal	whitish patches	antrum and angul	inflammation
31	Male	42	Reddish	Normal	whitish patches	antrum	inflammation
32	Male	42	Rough	Normal	Normal	antrum	inflammation
33	Male	47	Rough	Normal	Normal	antrum	intestinal metaplasia (WLE)
34	Male	40	Normal	Normal	Normal	antrum	intestinal metaplasia (random)
35	Male	70	Normal	Normal	Normal	antrum	intestinal metaplasia (random)
36	Male	71	Normal	Normal	Normal	antrum	intestinal metaplasia (random)
37	Male	41	Reddish	Normal	Normal	antrum and angul	inflammation
38	Male	40	Reddish	Normal	Normal	antrum	inflammation
39	Male	49	Reddish	Normal	Normal	antrum	inflammation
40	Male	63	Reddish	Normal	Normal	antrum	inflammation
41	Male	53	Reddish	Normal	Normal	antrum	inflammation
42	Male	47	Reddish	Normal	Normal	antrum	inflammation
43	Male	71	Rough	Normal	Normal	antrum	intestinal metaplasia (WLE)
44	Male	42	Rough	Normal	Normal	antrum	inflammation
45	Male	54	Rough	Normal	Normal	antrum and angul	inflammation
46	Male	51	Rough	Normal	Normal	antrum	inflammation
47	Male	45	Rough	Normal	Normal	antrum	inflammation
48	Male	40	Rough	Normal	Normal	antrum	inflammation
49	Male	50	Rough	Normal	Normal	antrum	inflammation
50	Male	43	Normal	Normal	Normal	antrum and angul	inflammation
51	Male	51	Normal	Normal	Normal	antrum and angul	inflammation
52	Male	42	Normal	Normal	Normal	antrum	inflammation
53	Male	46	Normal	Normal	Normal	antrum	inflammation
54	Female	40	Rough	bluish-whitish area	whitish patches	antrum and angul	intestinal metaplasia(AA-NBI)
55	Female	59	Whitish	bluish-whitish area	whitish patches	antrum and angul	intestinal metaplasia(AA-NBI,WLE)
56	Female	66	Whitish	bluish-whitish area	whitish patches	antrum	intestinal metaplasia(AA-NBI,WLE)
57	Female	58	Whitish	bluish-whitish area	whitish patches	antrum	intestinal metaplasia(AA-NBI,WLE)
58	Female	67	Whitish	bluish-whitish area	whitish patches	antrum	intestinal metaplasia(AA-NBI,WLE)
59	Female	69	Whitish	bluish-whitish area	whitish patches	antrum and angul	intestinal metaplasia(AA-NBI,WLE)
60	Female	46	Whitish	bluish-whitish area	whitish patches	antrum	intestinal metaplasia(AA-NBI,WLE)
61	Female	64	Reddish	bluish-whitish area	whitish patches	antrum	intestinal metaplasia(AA-NBI)
62	Female	69	Reddish	bluish-whitish area	whitish patches	antrum	intestinal metaplasia(AA-NBI)
63	Female	64	Reddish	bluish-whitish area	whitish patches	antrum	intestinal metaplasia(AA-NBI)
64	Female	50	Reddish	bluish-whitish area	whitish patches	antrum and angul	intestinal metaplasia(AA-NBI)
65	Female	60	Reddish	bluish-whitish area	whitish patches	antrum	intestinal metaplasia(AA-NBI)
66	Female	70	Reddish	bluish-whitish area	whitish patches	antrum	intestinal metaplasia(AA-NBI)
67	Female	66	Reddish	bluish-whitish area	whitish patches	antrum	intestinal metaplasia(AA-NBI)
68	Female	50	Rough	bluish-whitish area	whitish patches	antrum and angul	intestinal metaplasia(AA-NBI)
69	Female	60	Reddish	bluish-whitish area	whitish patches	antrum	intestinal metaplasia(AA-NBI,WLE)
70	Female	52	Reddish	bluish-whitish area	whitish patches	antrum	intestinal metaplasia(AA-NBI)
71	Female	75	Whitish	bluish-whitish area	whitish patches	antrum	inflammation
72	Female	68	Rough	bluish-whitish area	whitish patches	antrum	inflammation
73	Female	50	Rough	bluish-whitish area	whitish patches	antrum	inflammation
74	Female	44	Normal	bluish-whitish area	whitish patches	antrum and angul	inflammation
75	Female	41	Normal	Normal	whitish patches	antrum and angul	intestinal metaplasia(AA-NBI)
76	Female	42	Rough	Normal	whitish patches	antrum	inflammation
77	Female	40	Normal	Normal	whitish patches	antrum and angul	inflammation
78	Female	40	Normal	Normal	whitish patches	antrum and angul	intestinal metaplasia(AA-NBI)
79	Female	44	Normal	Normal	whitish patches	antrum	intestinal metaplasia(AA-NBI)
80	Female	51	Normal	Normal	whitish patches	antrum	intestinal metaplasia(AA-NBI)
81	Female	71	Normal	Normal	whitish patches	antrum	intestinal metaplasia(AA-NBI)
82	Female	42	Normal	Normal	whitish patches	antrum	intestinal metaplasia(AA-NBI)
83	Female	52	Reddish	bluish-whitish area	whitish patches	antrum	intestinal metaplasia(AA-NBI)
84	Female	45	Reddish	bluish-whitish area	whitish patches	antrum	intestinal metaplasia(AA-NBI)
85	Female	52	Reddish	bluish-whitish area	whitish patches	antrum	intestinal metaplasia(AA-NBI)
86	Female	57	Reddish	bluish-whitish area	whitish patches	antrum and angul	intestinal metaplasia(AA-NBI)
87	Female	46	Reddish	bluish-whitish area	whitish patches	antrum	intestinal metaplasia(AA-NBI)
88	Female	69	Reddish	bluish-whitish area	whitish patches	antrum	intestinal metaplasia(AA-NBI)
89	Female	59	Reddish	Normal	whitish patches	antrum	intestinal metaplasia(AA-NBI)
90	Female	51	Rough	Normal	whitish patches	antrum	intestinal metaplasia(AA-NBI)
91	Female	51	Normal	Normal	whitish patches	angulus	intestinal metaplasia(AA-NBI)
92	Female	47	Normal	Normal	whitish patches	antrum	intestinal metaplasia(AA-NBI)
93	Female	44	Normal	Normal	whitish patches	antrum	intestinal metaplasia(AA-NBI)
94	Female	65	Reddish	Normal	whitish patches	antrum	inflammation
95	Female	56	Reddish	Normal	whitish patches	antrum	inflammation
96	Female	51	Rough	Normal	whitish patches	antrum	inflammation
97	Female	69	Rough	Normal	whitish patches	antrum and angul	inflammation
98	Female	51	Rough	Normal	whitish patches	antrum	inflammation
99	Female	50	Normal	Normal	whitish patches	angulus	inflammation
100	Female	42	Normal	Normal	whitish patches	angulus	inflammation
101	Female	57	Normal	Normal	whitish patches	antrum	inflammation
102	Female	44	Reddish	Normal	Normal	antrum	inflammation
103	Female	51	Rough	Normal	Normal	antrum and angul	inflammation
104	Female	52	Normal	Normal	Normal	antrum	inflammation
105	Female	44	Normal	Normal	Normal	antrum and angul	intestinal metaplasia (random)
106	Female	71	Normal	Normal	Normal	antrum	intestinal metaplasia (random)
107	Female	73	Normal	Normal	Normal	antrum	intestinal metaplasia (random)
108	Female	44	Reddish	Normal	Normal	antrum	inflammation
109	Female	52	Reddish	Normal	Normal	antrum	inflammation
110	Female	66	Reddish	Normal	Normal	antrum	inflammation
111	Female	62	Reddish	Normal	Normal	antrum and angul	inflammation
112	Female	56	Reddish	Normal	Normal	antrum and angul	inflammation
113	Female	46	Reddish	Normal	Normal	antrum and angul	inflammation
114	Female	65	Reddish	Normal	Normal	antrum and angul	inflammation
115	Female	57	Reddish	Normal	Normal	antrum	inflammation
116	Female	80	Reddish	Normal	Normal	antrum	inflammation
117	Female	60	Reddish	Normal	Normal	antrum	inflammation
118	Female	60	Reddish	Normal	Normal	antrum	inflammation
119	Female	49	Reddish	Normal	Normal	antrum	inflammation
120	Female	40	Rough	Normal	Normal	antrum and angul	inflammation
121	Female	40	Rough	Normal	Normal	antrum	inflammation
122	Female	41	Rough	Normal	Normal	antrum	inflammation
123	Female	57	Rough	Normal	Normal	antrum	inflammation
124	Female	45	Normal	Normal	Normal	antrum	inflammation
125	Female	53	Normal	Normal	Normal	antrum	inflammation
126	Female	40	Normal	Normal	Normal	antrum and angul	inflammation
127	Female	57	Normal	Normal	Normal	angulus	inflammation
128	Female	46	Normal	Normal	Normal	antrum and angul	inflammation
129	Female	61	Normal	Normal	Normal	antrum	inflammation
130	Female	64	Normal	Normal	Normal	antrum	inflammation
131	Female	52	Normal	Normal	Normal	antrum	inflammation
132	Female	50	Normal	Normal	Normal	antrum and angul	inflammation
